# Supplementary material for: Mechanical Ventilation Strategies Targeting Different Magnitudes of Collapse and Tidal Recruitment in Porcine Acid Aspiration-Induced Lung Injury
Source: J Clin Med. 2019 Aug 18;8(8):1250. doi: 10.3390/jcm8081250 (PMC6722815; doi:10.3390/jcm8081250)
Supplement: Supplementary file 1 [file jcm-08-01250-s001.pdf]

Material and Methods

Animal Handling and Preparation

We studied German mixed country breed pigs aged 4–6 months with a mean body weight of 37 ± 5.2 kg. To obtain 24 animals for a per-protocol analysis, we had to enroll 27 pigs in our study. Two pigs developed massive pneumothoraces after intra-tracheal hydrochloric acid instillation (HCl, 0.1 mol, Rotipuran, Carl Roth GmbH, Karlsruhe, Germany) for induction of ARDS and died before randomization. One pig (EIT group) died eight hours after randomization because of intractable metabolic acidosis, which had developed immediately after instillation of HCl. Because a per-protocol analysis was planned an 'EIT' randomization envelope was added to compensate this dropout. Thus nine animals were randomized to the EIT group, but only the data of the eight animals, which completed the 24 h ventilation period, were analyzed (Figure 1 in main manuscript).

Pigs were fasted and premedicated with midazolam (1 $mg \cdot kg^{-1}$intramuscular) and ketamine (15 $mg \cdot kg^{-1}$intramuscular). Animals were anaesthetized by an infusion with fentanyl 5(3–30) $mg \cdot kg^{-1} \cdot h^{-1}$, midazolam 2 (1–6) $mg \cdot kg^{-1} \cdot h^{-1}$, ketamine 15 (5–30) $mg \cdot kg^{-1} \cdot h^{-1}$and pancuronium 0.15 $mg \cdot kg^{-1} \cdot h^{-1}$. After tracheotomy, animals were ventilated in supine position in volume-controlled mode via a 7-mm inner diameter endotracheal tube (Mallinckrodt, Athlone, Ireland) with a PEEP of 5 cm H_20, a tidal volume of 6 $mg \cdot kg^{-1}$of bodyweight and inspiratory-to-expiratory ratio = 1:2. Respiratory rate (RR) was adjusted to maintain pH > 7.30 (Evita XL, Dräger Medical Germany GmbH, Lübeck, Germany). Airway flow and pressure waveform signals were acquired continuously from the serial port of the ventilator. Peak ($P_{aw\text{-}peak}$) and plateau airway pressures ($P_{aw\text{-}plateau}$), as well as respiratory system compliance and driving pressure ($P_{aw\text{-}plateau}$ minus PEEP) were calculated using standard procedures [14]. $P_{aw\text{-}plateau}$ and PEEP were measured during zero flow conditions.

Mean arterial (MAP), heart rate (HR) and body temperature were recorded continuously (SC9000 monitor, Siemens, Erlangen, Germany). Sterile surgical instrumentation included an arterial catheter (5F 20 cm, Pulsion, Feldkirchen, Germany) in the femoral artery for pressure monitoring and continuous cardiac output (CO) measurement by transpulmonary thermodilution (PiCCO Plus Monitor, Pulsion Medical Systems, Feldkirchen, Germany). A central venous line (Certofix, Braun, Melsungen, Germany) was positioned in the left external jugular vein and a pulmonary artery catheter in the right one. Systemic vascular resistance (SVR) and pulmonary vascular resistance (PVR) were calculated. A urine catheter was surgically positioned in the urinary bladder (Coloplast, Humlebaek, Denmark).

All blood gas measurements (arterial and mixed-venous, CO-oximetry) were performed after 5 min of ventilation with pure oxygen (100%O_2) (ABL800, Radiometer Copenhagen, Copenhagen, Denmark).

After instrumentation, heparin was given as bolus of 15 $IE \cdot kg^{-1}$ and afterwards continuously infused at a rate of 200 IE per hour. Ringer-Acetat infusions with a rate of 5 $mL \cdot kg^{-1} \cdot h^{-1}$ were started after instrumentation. If the mean arterial pressure (MAP) decreased below 65 mmHg and/or diuresis decreased below 1 $mL \cdot kg^{-1} \cdot h^{-1}$, a 250 mL bolus of Ringer-Acetat solution was given if the stroke volume variation (SVV) greater than 10%. Otherwise noradrenaline infusion was started at a rate of 0.005 $\mu g \cdot kg^{-1} \cdot min^{-1}$ and subsequently titrated targeting a MAP of ≥ 65 mmHg. To prevent hemodynamic instability due to low right-ventricular pre-load resulting from high intrathoracic pressure during recruitment maneuvers, a 250 mL bolus of Ringer-Acetat was administered before recruitment.

Acid Aspiration Induced Lung Injury

Pulmonary ARDS was induced by intra-tracheal hydrochloric acid instillation (HCl, 0.1 molar, Rotipuran, Carl Roth GmbH, Karlsruhe, Germany). During instillation of hydrochloric acid, the breathing circuit was disconnected and a 16 Charriere suctioning catheter was advanced 30 cm via the endotracheal tube until its tip had advanced 5 cm beyond the end of the endotracheal tube. A marking had been made on the catheter prior to insertion to ensure standardized insertion length.

After placement of the catheter 30 mL of HCl were injected into the trachea. Afterwards the catheter was withdrawn so that its tip was positioned at the tip of the tube and another 30 mL of HCl were installed.

Computed Tomography (CT)

Helical CT scans (Philips MX8000 IDT 16 (120-kV tube voltage, 170-mA tube current, 16 × 1.5-mm collimation; Philips Medical System, Hamburg, Germany) were acquired at baseline (BL), ARDS installation (ARDS) and further every 4 h afterwards until 24 h of mechanical ventilation were achieved. CT scans were taken in 3 mm and reconstructed with the standard filter "B" (MX8000 IDT 16, 120-kV tube voltage, 170-mA tube current, 16 × 1.5-mm collimation; Philips Medical Systems, Hamburg, Germany) in 6mm slice thickness without any contrast medium. Window level and width (level/width) were set to −500/1500 Hounsfield Units (HU) for displaying lung parenchyma on the monitor of the CT-processing computer and level/width of 50/250 HU were used for the mediastinum and soft tissues. Segmentation of the lung parenchyma and exclusion of major vascular and bronchial structures was performed manually based on anatomic knowledge (Osiris software, University Hospital of Geneva, Switzerland). Partial volume effects were minimized using a cutoff value of −350 HU in aerated lung regions [28,51,52]. Small negative numbers as results of tidal recruitment (TR) calculation (4 measurements) were explained by inter-observer differences in the segmentation of CT images (i.e., up to 3%) [53] and were set to zero.

Quantitative Computed Tomography (qCT)

Whole-lung CT scans were taken without contrast medium. After manual segmentation of CT images, qCT-parameters were calculated using standard procedures. Total lung mass (M_{total}) and volume (V_{total}) were identified within [−1000 to 100] Hounsfield units (HU). Differently aerated lung compartments were defined by the following intervals and calculated as percentage of M_{total}: hyperinflated (M_{hyper}, [−1000 to -901] HU), normally aerated (M_{normal}, [-900 to -501] HU), poorly aerated (M_{poorly}, [-500 to −101] HU) and non-aerated (M_{non}, [−100 to 100] HU) [27–30]. We calculated TR by subtracting inspiratory from expiratory M_{non}. To characterize the time-weighted cumulative impact which different amounts of TR had on the lung parenchyma, we conceived the surrogate 'tidal-recruitment-hours' (TR-hours): We multiplied the amount of TR at a given measurement point by the period it had acted since the last measurement. These products from all measurement points were then summed up to give TR-hours.

Electrical Impedance Tomography

EIT data were recorded using Pulmo Vista 500 EIT system (Dräger Medical Germany, GmbH, Lübeck, Germany). The EIT data for calculating the regional ventilation delay (RVD) as surrogate for inhomogeneous regional ventilation was recorded during a slow inflation maneuver (inspiratory flow 4 $L \cdot min^{-1}$, inflation volume 12 $mL \cdot kg^{-1}$). After transferring this EIT data to a personal computer equipped with customized software [23], the RVD and the standard deviation over all single pixel's RVD (SD_{RVD}, to quantify the RVD inhomogeneity) were calculated by offline analysis [23]. In analogy to TR-hours, SD_{RVD}-hours were calculated multiplying SD_{RVD}. In addition, the "center of ventilation" was used to quantify differences in the regional distribution of ventilation [31]. For EIT parameter analysis, mean tidal images over 40 s are reconstructed using Dräger algorithms for each animal and point in time. Percentages for ventral and dorsal distribution of ventilation (positive pixel values) in EIT images are calculated based on those tidal images, which are averaged over each group at each point in time for the visualization (Figure 4 in main manuskript).

Tissue Processing and Histological Analysis

After the end of the study, animals were killed by injection of 50 mmol KCl under increased anesthesia. The lung and heart were removed en bloc occurred under continuous airway pressure set to the plateau pressure of the last ventilation period. The left lung was fixed by perfusion with

formaldehyde solution 4% through a catheter inserted in the left main pulmonary artery for at least one hour. Before starting the perfusion, the lung vasculature was flushed with one liter of Ringer-Acetat solution. During the flushing and perfusion fixation procedure, the perfusion pressure was monitored and adjusted to match the last measured mean pulmonary artery pressure and the lung was kept inflated using the last end-expiratory pressure (PEEP) [14]. After fixation, four standardized tissue samples (ventral, medio-ventral, medio-dorsal and dorsal) following gravitational changes from the cranial left lower lobe were taken (6–8 cm^3). The left lung was chosen to limit the probability that the histological changes detected originate predominantly from direct contact with the hydrochloric acid, as could be expected for the lower lobe of the right lung. Fixation was continued in 4% buffered formaldehyde solution for 24 h. Dehydration and paraffin fixation for 48 h was performed using an automatic tissue processor (Histokinette, Medim DDM-P-801, Medim Switzerland GmbH, Baar, Switzerland). Afterwards tissue samples were embedded in paraffin at 60 °C, cut into 5 μm slices and stained with hematoxylin-eosin. Microscopic images from a light microscope (Zeiss Axio Imager with Axio Cam MRc5, Carl Zeiss AG, Feldbach, Switzerland) for analysis were taken in two different magnifications (100×, 400×) from four non-overlapping fields of view per slide. Image processing was performed using dedicated software (ZeissAxio Vision Rel. 4.8). Three different observers, who were blinded to the ventilation protocols, accessed the criteria hemorrhage, inflammation and intra-alveolar edema of the Diffuse Alveolar Damage (DAD) score [33,34]. The scores read by the three independent observers showed a strong Intra Class Correlation (ICC = 0.88). For each criterion the score could be between zero and 12, increasing in degree of damage. Values from 0 to 3 represented the severity of the feature (0: normal appearance, 1: minor effect, 2: moderate effect and 3: severe effect) and were multiplied by the amount of involvement in each field of view (0: no presence of the criterion, 1: up to 25% involvement, 2: 26–50%, 3: 51–75%, 4: 76% to total involvement of the field). Because histological analysis detected no meaningful or significant differences between tissue samples from different lung regions (Table S3), we decided to average regional scores into single global DAD scores for each histological criterion [14,34]. We calculated the cumulative Diffuse Alveolar Damage (DAD) Score as the sum over all criteria [33,34] using the global scores as well.

Table S3. Regional results in histological analysis.

	DAD score				Global DAD score	*P-value (Friedman)*
	**ventral**	**medial-ventral**	**medial-dorsal**	**dorsal**		
edema						
ARDSnet	2.9± 1.9	4.1± 3.2	3.1± 2.3	4.2± 2.6	3.6±1.7	0.58
OLC	1.2± 2.1	1.6± 0.8	2.2± 1.4	2.3± 1.9	1.8±1.0	0.08
EIT	1.9± 1.4	2.2±2.1	1.8± 1.7	2.4± 1.4	2.1±1.4	0.54
P-value (Kruskal-Wallis)	0.06	0.35	0.41	0.28		
inflammation						
ARDSnet	3.6± 1.9	4.2± 2.1	3.6± 1.3	5.6± 2.3	4.3±1.2	0.24
OLC	1.7± 1.9	2.4± 1.3	2.7± 1.3	3.9± 3.3	2.7±1.2	0.47
EIT	3.7± 2.1	3.8± 2.2	3.4± 2.1	4.3± 1.0	3.8±1.1	0.68
P-value (Kruskal-Wallis)	0.03	0.19	0.59	0.36		
hemorrhage						
ARDSnet	2.1± 1.7	3.1± 2.8	1.7± 1.5	2.1± 1.9	2.2±1.7	0.45
OLC	1.5± 2.7	1.8±1.8	1.7± 1.2	2.3± 1.9	1.8±1.4	0.07
EIT	2.5± 2.0	3.9± 2.2	2.4± 1.9	2.3± 1.1	2.5±1.4	0.48
P-value (Kruskal-Wallis)	0.64	0.37	0.69	0.83		

ARDSnet, OLC and EIT denote the treatment groups. The criteria edema, hemorrhage, and inflammation were analyzed in tissue samples taken from ventral, medial-ventral, medial-dorsal and dorsal lung regions according to the diffuse alveolar damage scores (DAD score) read by three blinded observers [33]. Data are presented as mean and standard deviation. The use of mean and standard deviation results from averaging the results of the three readers. Because of significant deviations from normal distribution, nonparametric tests (Friedman or Kruskal-Wallis) were preferred for between- and within-group analyses. Except for the between-group difference for inflammation in ventral lung regional, whose significance is interpreted with caution because of multiple testing, we could not detect any significant differences in regional distribution of histological.

Wet-to-dry ratio

One tissue sample from the accessory lung lobe was taken. This tissue was immediately weighed and, after drying it for 72 h at 50 °C, it was weighed again. The wet-to-dry ratio was calculated as the ratio between these two measurements [54].

Statistical Analysis

We found only one study reporting suitable data for sample size estimation [55]. From the results of this previous study, we assumed a mean cumulative DAD = 10 (SD = 2) points for the criteria hemorrhage, inflammation and intra-alveolar edema, for our ARDSnet and OLC groups. Hypothesizing that the DAD would be one point lower for each DAD criterion in the EIT group, at least seven animals were required per group (alpha = 0.05, beta = 0.2) (KaneSP). We increased this number to eight animals per group to compensate for potential differences in histological analyses due to different ARDS models. Depending on normal distribution (investigation of D'Agostino and Pearson's tests, dot plots and QQ-plots), data is presented as mean with its standard deviation (SD) or 95% confidence interval. Changes from BL to established ARDS (all animals before randomization) were analyzed using paired t-tests. General Linear Model (GLM) approaches were used to analyze group, time and interactions effects for repeated measurements as well as for univariate comparisons. If sphericity could not be demonstrated (Mauchly's test), Greenhouse-Geisser-corrected P-values are reported. Because GLM analyses are quite robust against minor deviations from normal distribution and compatible nonparametric statistical techniques are not available, we used the GLM approach also for within- and between-group analyses of data with minor deviations from normal distribution, such as TR (Table S1). Only if the GLM indicated significant differences, Sidak's post-hoc tests were performed. Regional histological scores were analyzed by nonparametric tests [33,56,57] because of significant deviations from normal distribution. After establishing global DAD scores following normal distribution analysis of variance (ANOVA) was performed. Correlation between DAD scores of the three observers was tested using Intra Class Correlation (ICC) [58]. Statistical software SPSS 22 (SPSS Inc., Munich, Germany) and GraphPad Prism 5 (GraphPad Software Inc., La Jolla, CA, USA) was used. We considered *P*-values below 0.05 significant.

Results

Systemic physiological parameters

Table S2. Body temperature and fluid balance.

	Group	Baseline	ARDS	4 hrs	8 hrs	12 hrs	16 hrs	20 hrs	24 hrs	Time effect	Inter-action	Group effect
**Body temperature (°C)**	ARDSnet<br>OLC<br>EIT	36.5 ± 1.2	36.2 ± 1.5<br>36.9 ± 1.4<br>36.7 ± 0.6	38.5 ± 1.2<br>38.5 ± 1.8<br>37.8 ± 1.8	39.5 ± 1.1<br>39.7 ± 1.5<br>38.1 ± 1.8	40.1 ± 0.8<br>39.7 ± 1.8<br>38.5 ± 1.8	39.9 ± 1.3<br>39.7 ± 1.6<br>38.5 ± 1.8	38.6 ± 1.3<br>40.0 ± 1.2<br>38.6 ± 1.8	38.6 ± 0.8<br>40.4 ± 1.0<br>39.5 ± 1.9	P_1 0.59<br>$P_2 < 0.001$	0.12	0.11
**Fluid balance (ml·kg⁻¹ BW)**	ARDSnet<br>OLC<br>EIT	25.8 ± 17.2	33 ± 29.7<br>19 ± 23.1<br>42 ± 31.9	48 ± 29.2<br>39 ± 34.7<br>101 ± 56.9	63 ± 30.1<br>64 ± 47.5<br>124 ± 48.6	75 ± 34.1<br>82 ± 55.3<br>138 ± 38.8	77 ± 29.5<br>107 ± 61.5<br>162 ± 35.8	84 ± 34.9<br>125 ± 74.5<br>189 ± 40.5	111 ± 64.4<br>131 ± 68.9<br>211 ± 44.7	P_1 0.47<br>$P_2 < 0.001$	0.005	0.03<br>A = 1.0<br>B = 0.048<br>C = 0.067

The table specifies changes of body temperature, measured by the thermistor of the PiCCO system in the aorta, and the cumulative fluid balance in ml·kg^{-1} bodyweight (BW). Data are presented in mean with standard deviation. ARDSnet, OLC, and EIT denote the treatment groups. Because randomized group allocation happened only after induction of ARDS, baseline measurements are averaged over all animals. P_1 indicates statistically significant differences between baseline and ARDS. This was analyzed by paired t-tests over all animals (*n* = 24), irrespective of the group. P_2 marks parameters, for which the GLM statistics over all measurements from ARDS until 24 h indicated significant changes over time. Furthermore the columns interaction effect and group effect marks parameters for which the GLM indicated significant interaction terms (group×time) and significant between-group differences. These are statistically calculated with Sidak's posthoc test and results are represented with A (refers to ARDSnet-vs.-OLC), B (refers to ARDSnet-vs.-EIT) and C (refers to OLC-vs.-EIT). $P < 0.05$ was considered significant. Asterisks are indicating statistical significance: one asterisk refers to $P < 0.05$, two asterisks to $P < 0.01$, and three asterisks to $P < 0.001$.

Respiratory Parameters

Quantitative Computed Tomography (qCT) and EIT parameters

Contrary to M_{non}, M_{normal}, M_{poor}, gas content, M_{total} (all $P < 0.001$) and V_{total} ($P = 0.01$), which all changed significantly from BL after induction of ARDS, M_{hyper} ($P = 0.18$) was not changed by induction of ARDS (Figure 2, Figure S2, Table S1).

M_{hyper} increased over time (time effect $P = 0.001$) but with no significant differences between groups ($P = 0.17$) or interaction ($P = 0.10$). The M_{poor} also changed significantly over time ($P = 0.03$), differed between groups ($P < 0.001$) and showed an interaction effect ($P < 0.001$) with differences between ARDSnet and EIT ($P < 0.001$) and ARDSnet-vs.-OLC ($P = 0.004$) but not between OLC and EIT ($P = 0.32$). M_{normal}, V_{total} and gas content all showed the same behavior with significant changes in time ($P < 0.001$), group ($P < 0.001$) and interaction ($P < 0.001$). M_{total} increased in all groups over time ($P < 0.001$) without a statistically significant difference between groups ($P = 0.12$) or interaction ($P = 0.06$). Changes in Mnon are presented in Figure 2 and in Table S1.

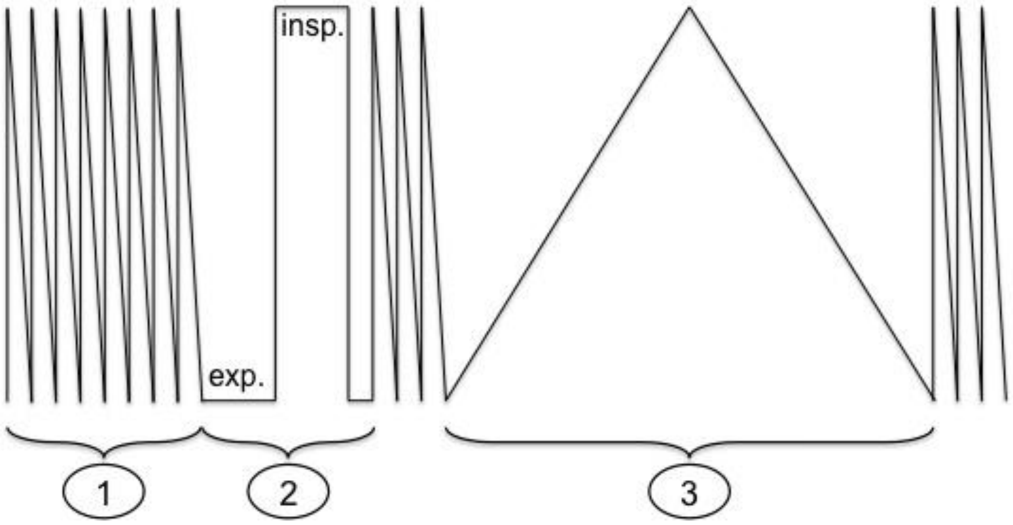

Figure S1. Schematic presentation of the standardized sequence of data acquisition at each measurement point. (1) Acquisition of cardiorespiratory, ventilatory, hemodynamic and tidal EIT data, (2) CT- acquisition during inspiration (insp) and expiration (exp) and (3) recording of EIT data during the slow inflation maneuver.

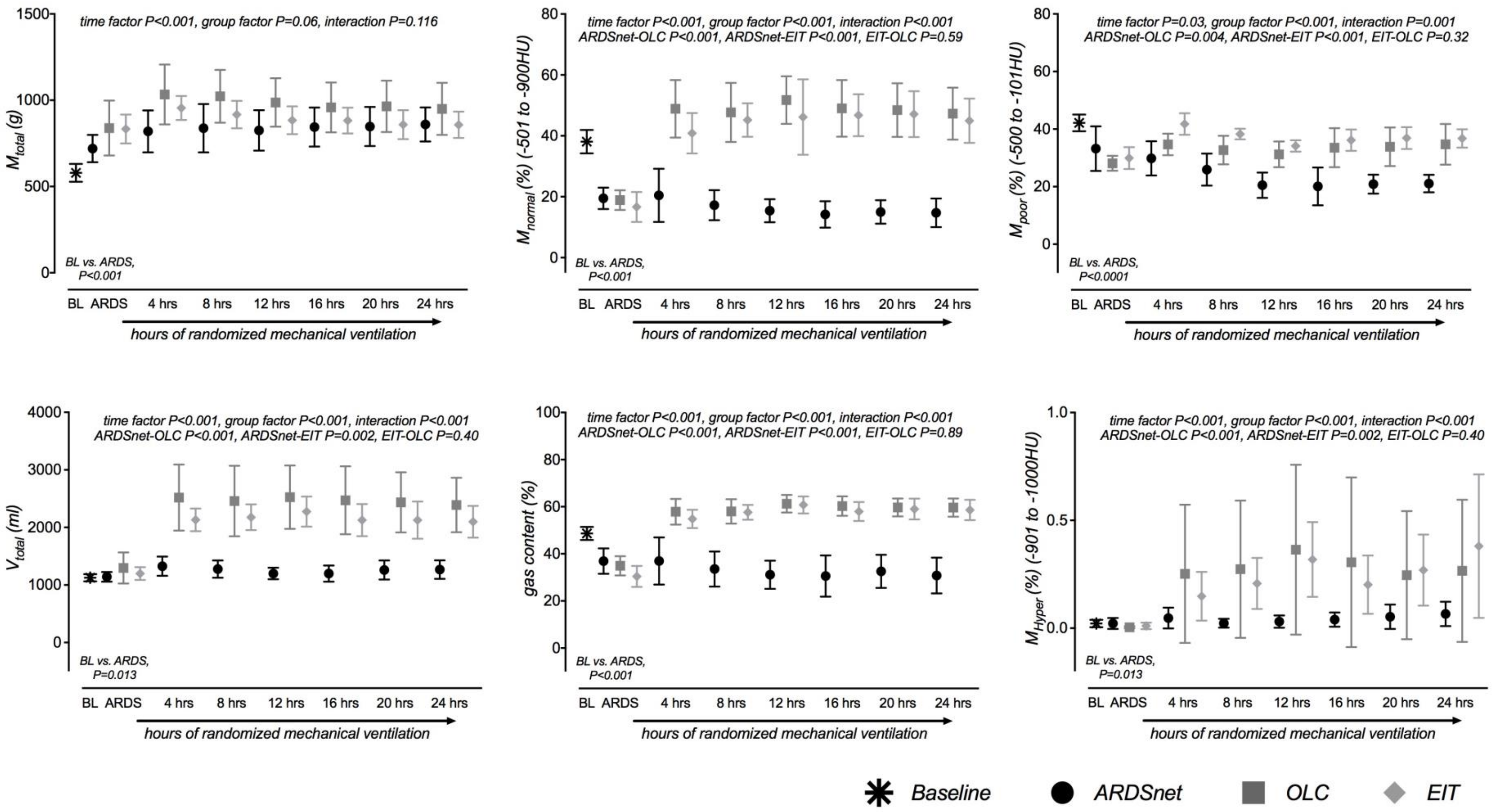

Figure S2. Quantitative CT parameters. Measurements were performed at baseline (BL), ARDS/randomization (ARDS) and every 4 h for 24 h for the three ventilation groups ARDSnet, Open Lung Concept (OLC), and Electrical Impedance Tomography (EIT). Data of poor aerated lung tissue mass (Mpoor), normal aerated lung tissue (Mnormal), hyper aerated lung tissue (Mhyper) and gas content are shown in percent, total lung mass (Mtotal) in g and total lung volume (Vtotal) in ml. Data are shown as mean and its 95% confidence intervals. Differences between BL and ARDS were tested with paired t-test without groups. Time and group effects and their interaction were tested using the general linear model approach. Differences between ventilation groups were tested by Sidak's post-hoc test.

Table S1. Changes in quantitative Computed Tomography (qCT) and Electrical Impedance Tomography (EIT) parameters.

.	Group	Baseline	ARDS	4h	8h	12h	16h	20h	24h	Time Effect	Inter-action	Group Effect
**M total (g)**	ARDSnet		719.9 ± 94.8	819.5 ± 145.3	837.7 ± 167.5	825.0 ± 139.7	838.5 ± 144.9	847.4 ± 135.5	859.4 ± 117.9			
	OLC	579.1 ± 122.2	838.7 ± 190.5	1033 ± 207.5	1025 ± 197.4	987.0 ± 168.0	958.4 ± 172.7	964.4 ± 178.3	949.9 ± 180.3	**P_1 *** P_2 *****	**0.06**	
	EIT		833.2 ± 99.6	955.3 ± 83.0	916.9 ± 95.0	883.9 ± 96.5	881.8 ± 89.4	858.3 ± 100.0	857.5 ± 91.9			
**V total (mL)**	ARDSnet		1141 ± 99.4	1326 ± 199.0	1277 ± 179.6	1199 ± 119.2	1198 ± 167.3	1260 ± 199.0	1268 ± 191.6			**a *****
	OLC	1125.6 ± 147.8	1296 ± 323.3	2518 ± 685.7	2515 ± 771.8	2593 ± 681.1	2472 ± 705.5	2436 ± 625.2	2389 ± 565.8	**P_1 0.013 P_2 *****	**P *****	**b 0.002**
	EIT		1199 ± 133.4	2131 ± 235.7	2177 ± 267.5	2275 ± 312.9	2127 ± 333.4	2127 ± 386.2	2099 ± 329.8			**c 0.397**
**M_{poor} (%) [−500 to −101HU]**	ARDSnet		33.2 ± 9.3	29.8 ± 7.1	25.9 ± 6.7	20.5 ± 5.3	20.1 ± 7.9	20.9 ± 3.9	21.1 ± 3.6			**a 0.004**
	OLC	42.2 ± 6.9	28.1 ± 3.1	29.7 ± 14.0	32.7 ± 5.9	31.2 ± 5.3	33.5 ± 8.1	33.9 ± 8.0	34.7 ± 8.4	**P_1 *** P_2 0.025**	**P *****	**b *****
	EIT		29.9 ± 4.5	41.8 ± 4.5	38.3 ± 2.2	34.1 ± 2.4	36.1 ± 4.4	36.9 ± 4.5	36.8 ± 3.8			**c0.317**
**M_{normal} (%) [−501 to −950HU]**	ARDSnet		19.5 ± 4.2	20.5 ± 10.4	17.2 ± 5.9	15.4 ± 4.5	14.2 ± 5.2	15.0 ± 4.6	14.7 ± 5.6			**a *****
	OLC	38.1 ± 9.1	18.9 ± 3.9	48.9 ± 11.3	47.7 ± 11.6	51.7 ± 9.3	49.0 ± 11.1	48.5 ± 10.5	47.3 ± 10.2	**P_1 *** P_2 *****	**P *****	**b *****
	EIT		16.6 ± 5.9	40.8 ± 7.9	45.2 ± 6.6	46.2 ± 14.8	46.8 ± 8.2	47.1 ± 9.0	45.0 ± 8.7			**c 0.589**
**M_{hyper} (%) [−951 to −1000HU]**	ARDSnet		0.02 ± 0.03	0.47 ± 0.58	0.02 ± 0.02	0.03 ± 0.03	0.04 ± 0.04	0.05 ± 0.07	0.07 ± 0.07			
	OLC	2.2 ± 4.1	0.004 ± 0.01	0.25 ± 0.41	0.28 ± 0.41	0.36 ± 0.47	0.31 ± 0.47	0.25 ± 0.36	0.27 ± 0.4	**P_1 0.181 P_2 0.001**	**0.098**	
	EIT		0.01 ± 0.02	0.15 ± 0.14	0.21 ± 0.14	0.31 ± 0.21	0.20 ± 0.16	0.27 ± 0.20	0.38 ± 0.40			
**Gas content (%)**	ARDSnet		36.9 ± 6.5	36.9 ± 12.0	33.5 ± 8.9	31.1 ± 7.1	30.6 ± 10.5	32.5 ± 8.4	30.8 ± 9.1			**a *****
	OLC	48.7 ± 6.5	34.9 ± 4.9	57.9 ± 6.5	58.0 ± 6.2	61.3 ± 4.5	60.3 ± 4.9	59.7 ± 4.5	59.6 ± 4.6	**P_1 *** P_2 *****	**P *****	**b *****
	EIT		30.4 ± 5.3	54.8 ± 4.6	57.6 ± 3.7	60.8 ± 4.3	58.0 ± 4.8	59.0 ± 5.3	58.6 ± 5.1			**c 0.895**
**M_{non} (%) [−100 to 100HU]**	ARDSnet		47.3 ± 11.1	50.3 ± 14.0	57.8 ± 11.6	64.4 ± 8.5	66.1 ± 10.3	63.2 ± 6.6	63.4 ± 7.0			**a *****
	OLC	19.8 ± 11.8	53.0 ± 4.3	16.2 ± 10.3	19.3 ± 13.4	16.7 ± 11.3	17.1 ± 11.7	17.5 ± 12.6	17.7 ± 12.3	**P_1 *** P_2 *****	**P *****	**b *****
	EIT		54.9 ± 5.7	15.8 ± 6.1	15.2 ± 6.2	14.9 ± 6.2	17.0 ± 5.6	15.9 ± 5.5	17.5 ± 6.0			**c 0.99**
**SD_{RVD}**	ARDSnet		8.0 ± 2.7	8.2 ± 3.2	9.2 ± 3.3	9.4 ± 3.0	9.8 ± 2.3	8.6 ± 2.3	7.6 ± 2.9			**a 0.003**
	OLC	4.5 ± 2.5	6.4 ± 3.0	4.9 ± 1.8	4.9 ± 1.2	4.3 ± 0.9	5.1 ± 1.5	4.8 ± 0.9	5.9 ± 3.0	**P_1 *** P_2 0.004**	**P 0.002**	**b 0.015**
	EIT		10.5 ± 3.4	4.5 ± 1.6	4.3 ± 1.7	4.7 ± 1.5	5.2 ± 2.2	5.1 ± 2.4	5.5 ± 1.9			**c 0.829**
**CoV (%)**	ARDSnet		56.0 ± 3.7	56.0 ± 4.2	56.8 ± 2.9	57.7 ± 2.2	57.3 ± 4.1	57.1 ± 2.7	56.9 ± 3.2			**a 0.01**
	OLC	55.8 ± 2.5	56.4 ± 2.0	52.6 ± 1.6	52.0 ± 2.0	51.5 ± 2.0	51.9 ± 1.7	50.7 ± 3.2	49.8 ± 4.5	**P_1 0.302 P_2 *****	**P *****	**b 0.001**
	EIT		56.7 ± 3.0	50.8 ± 2.3	50.6 ± 3.0	49.9 ± 3.2	50.8 ± 1.8	51.1 ± 2.6	51.5 ± 2.4			**c 0.69**

ARDSnet, OLC and EIT denote the treatment groups. Total lung mass (M_{total}), total lung volume (V_{total}), percentage of nonaerated lung mass (M_{non}), percentage of poorly aerated lung mass M_{poor}), percentage of normally aerated lung mass (M_{normal}), percentage of hyperaerated lung mass (M_{hyper}), percentage of gas volume of the total lung volume (gas content), standard deviation of regional ventilation delay (SD_{RVD}), and Center of Ventilation (CoV) are given as mean and standard deviation. Because randomized group allocation happened only after induction of ARDS, baseline measurements are averaged over all animals. P_1 indicates statistically significant differences between baseline and ARDS, this was analyzed by paired t-tests over all animals (*n* =2 4), irrespective of the group. P_2 marks parameters, for which the GLM statistics over all measurements from ARDS until 24 h indicated significant changes over time. Furthermore the columns interaction effect and group effect marks parameters, for which the GLM indicated significant interaction terms (group×time) and significant between-group differences. These are statistically calculated with Sidak’s posthoc test and results are represented with A (refers to ARDSnet-vs.-OLC), B (refers to ARDSnet-vs.-EIT) and C (refers to

OLC-vs.-EIT). $P < 0.05$ was considered significant. Asterisks are indicating statistical significance: one asterisk refers to $P < 0.05$, two asterisks to $P < 0.01$, and three asterisks to $P < 0.001$. According to the D'Agostino & Pearson omnibus normality test, TR was not normally distributed. TR data are shown as mean with SD as well. Because inspection of dot plots and Q–Q plots for tidal recruitment data showed, however, that deviation from normal distribution was minor, we decided to still use the GLM approach to analyze within- and between group difference, because no non-parametric alternative to such GLM analysis is available.

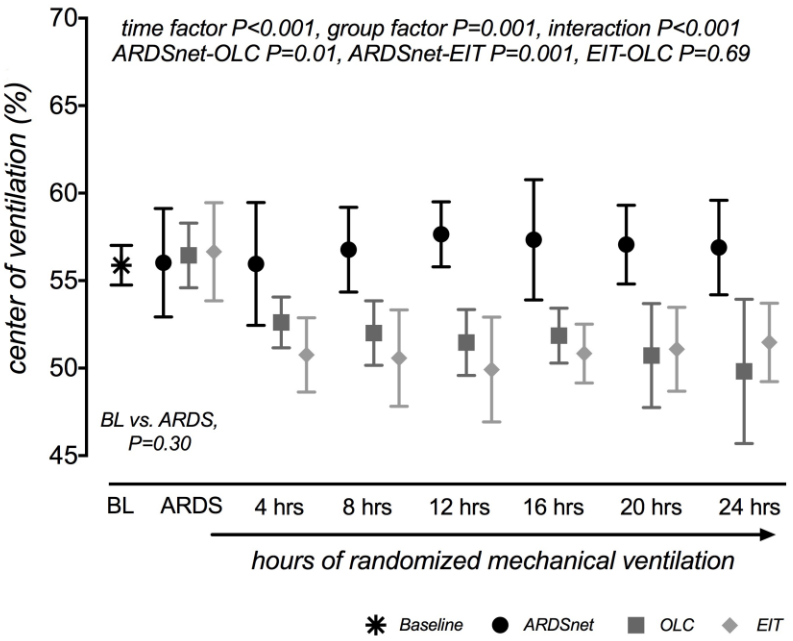

Figure S3. Center of Ventilation (CoV). The EIT-derived center of ventilation is shown as a surrogate of the distribution of ventilation on the ventro-dorsal lung diameter. The CoV is given in percent (%), 100% corresponding to the most ventral and 0% to the most dorsal lung regions. Measurements were performed at baseline (BL), ARDS/randomization (ARDS) and every 4 h for 24 h for the three ventilation groups ARDSnet, Open Lung Concept (OLC), and Electrical Impedance Tomography (EIT). Data are shown as mean with standard deviation. Time effect was tested by general linear model. Differences between ventilation groups were tested by Sidak's post hoc test.

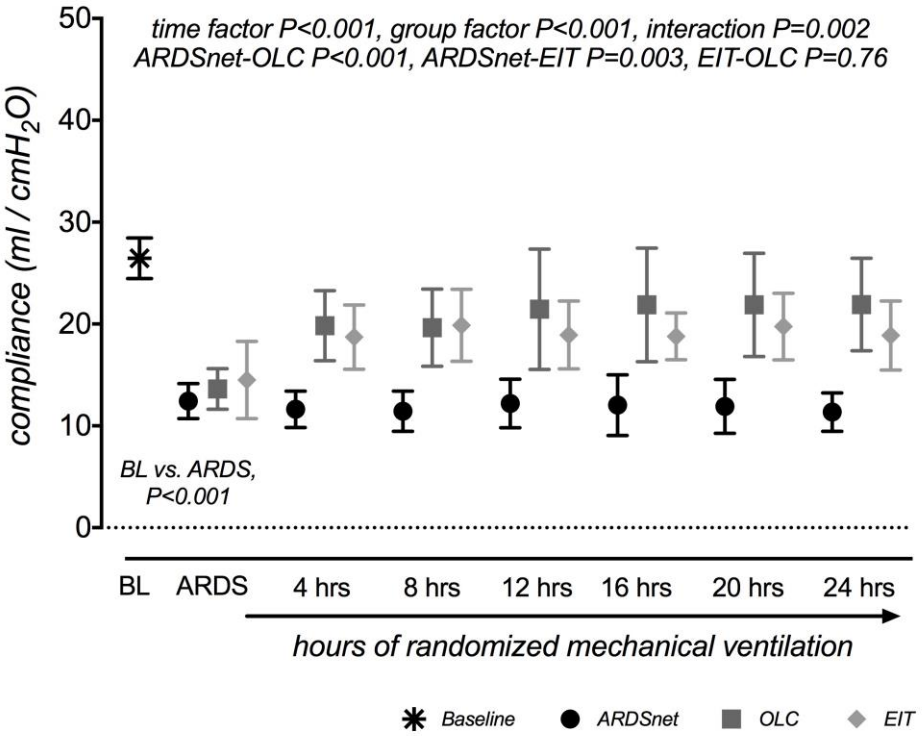

Figure S4. System compliance. Within- and between-group changes of respiratory system compliance (measured during zero-flow conditions) over 24 h in the three different ventilation groups ARDSnet, OLC, and EIT. Symbols and error bars are means with 95% confidence intervall. Changes occuring between baseline (BL) and ARDS/randomization (ARDS) before group allocation were tested with paired t-tests. Within- and between-group changes occuring after randomization (column ARDS) were tested using a General Linear Model approach together with Sidak's post-hoc test.

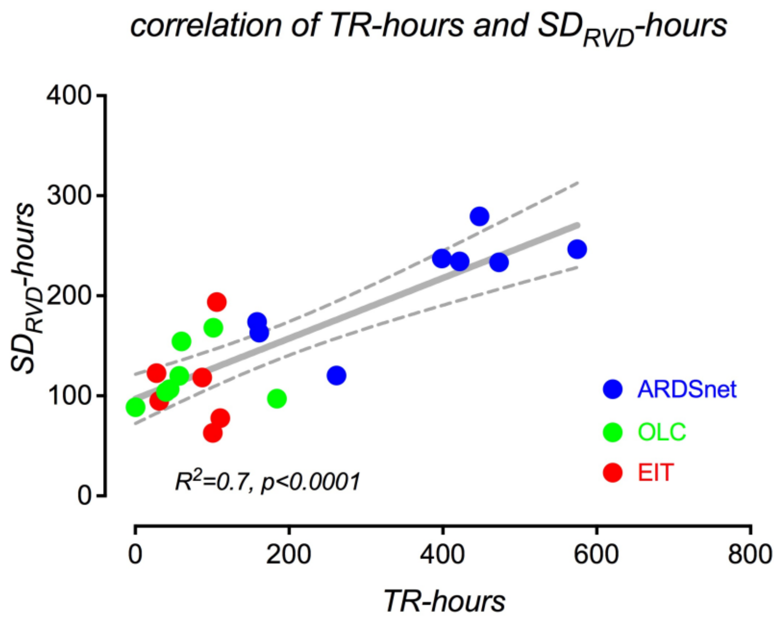

Figure S5. Correlation of tidal-recruitment-hours (TR-hours) and SD_{RVD}-hours. TR-hours and SD_{RVD}-hours (SD_{RVD} = standard deviation of all pixels regional ventilation delay) showing a significant correlation over all animals (linear regression, $R^2 = 0.7$, $P < 0.001$, regression line is presented with error bars). The SD_{RVD}-hours were calculated as followed: (1) SD_{RVD} determined at a certain measurement point was calculated by the time elapsed since the last measurement, (2) the sum over all these products gave the total SD_{RVD}-hours. As a consequence of the underlying physiological concept, pigs in different groups showed different amounts of TR or SD_{RVD}.

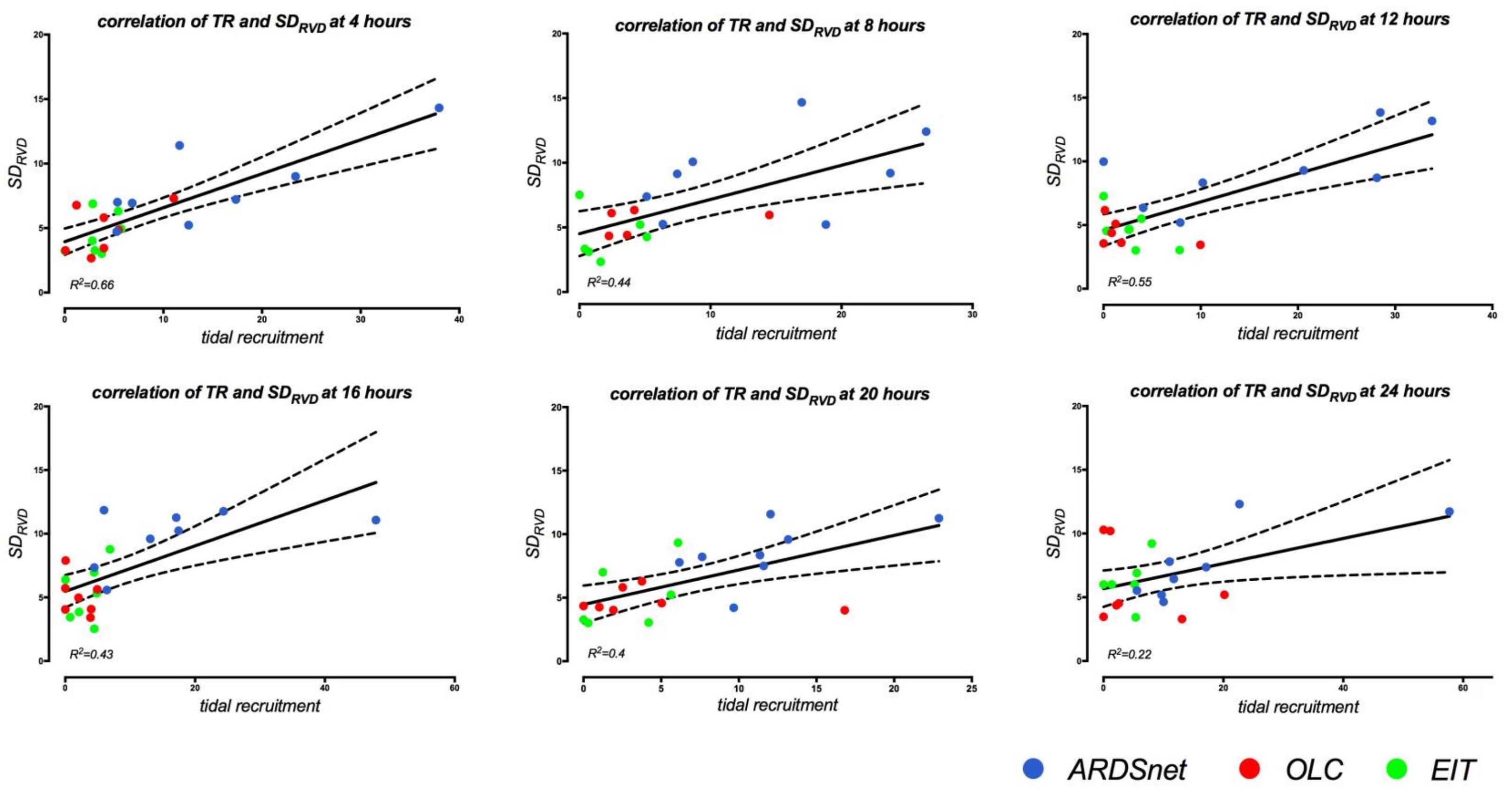

Figure S6. Correlation of TR and SD_{RVD} at different measurement points throughout the trial. Noticing a tendency towards correlation especially with higher signals in ARDSnet group for TR and SD_{RVD}, we thought of a possibility to include the temporal aspect and the summed impact of TR/SD_{RVD} to the lung creating TR-/SD_{RVD}-hours as mentioned above. Linear regression (R^2) has been used for analysis and is presented with regression line and error bars in the graphics.

Histological Results

Surrogates of tidal recruitment derived from quantitative CT or EIT damage for each ventilation group. Therefore a global DAD Score out of all regional DAD scores per group was created.

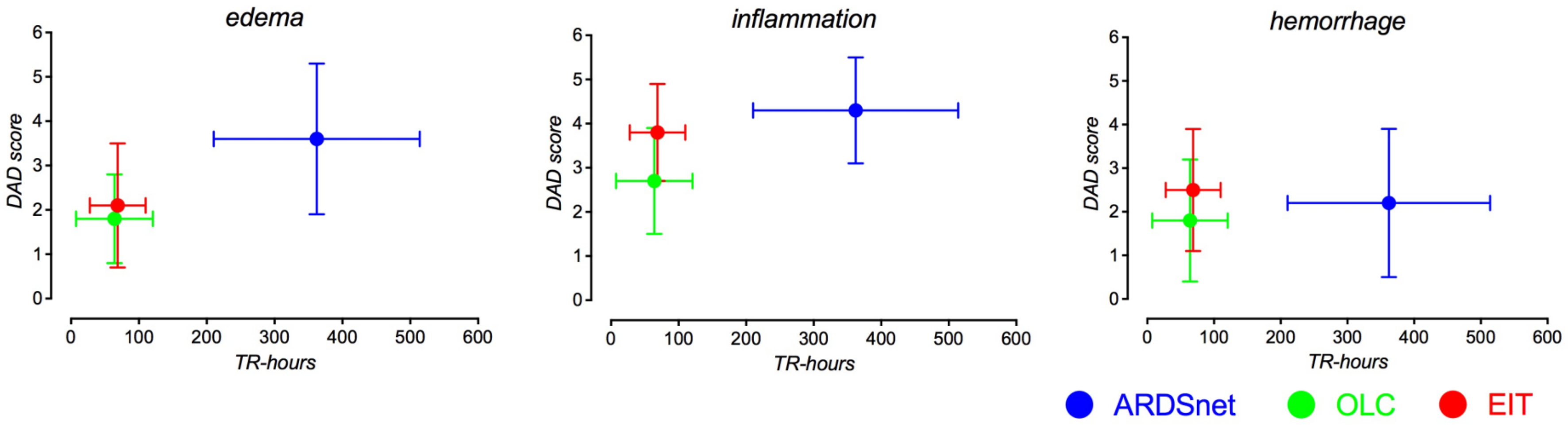

Figure S7. Association of the global DAD score and its histological criteria with the magnitude of tidal recruitment expressed as tidal-recruitment-hours (TR-hours). Data are presented as Mean plus error bars with standard deviation. For edema and inflammation, the OLC and EIT strategies showed lower scores in histological damage and reduced TR. Hemorrhage did not differ between groups in histology. The magnitude of TR differed significantly between groups ($P < 0.001$), ARDSnet group (362 ± 152 TR-hours), EIT (67 ± 38 TR-hours) and OLC (64 ± 57 TR-hours).
